# Transcriptional and post-transcriptional control of epithelial-mesenchymal plasticity: why so many regulators?

**DOI:** 10.1007/s00018-022-04199-0

**Published:** 2022-03-12

**Authors:** Melodie Migault, Sunil Sapkota, Cameron P. Bracken

**Affiliations:** 1 Gene Regulatory Networks Laboratory, Centre for Cancer Biology, an Alliance between the University of South Australia and SA Pathology, Bradley Building, Rm HB-9-31, North Terrace, Adelaide, SA 5000 Australia; 2 Division of Medicine, University of Adelaide, Adelaide, SA Australia; 3 School of Life Sciences, University of Adelaide, Adelaide, SA Australia

**Keywords:** Epithelial-mesenchymal transition, Transcription factor, microRNA, Cell signaling, Gene networks, Cancer

## Abstract

The dynamic transition between epithelial-like and mesenchymal-like cell states has been a focus for extensive investigation for decades, reflective of the importance of Epithelial-Mesenchymal Transition (EMT) through development, in the adult, and the contributing role EMT has to pathologies including metastasis and fibrosis. Not surprisingly, regulation of the complex genetic networks that underlie EMT have been attributed to multiple transcription factors and microRNAs. What is surprising, however, are the sheer number of different regulators (hundreds of transcription factors and microRNAs) for which critical roles have been described. This review seeks not to collate these studies, but to provide a perspective on the fundamental question of whether it is really feasible that so many regulators play important roles and if so, what does this tell us about EMT and more generally, the genetic machinery that controls complex biological processes.

## Introduction

Epithelial-Mesenchymal Transition (EMT) describes a process by which epithelial cells, possessing apical-basal polarity and characterized by stable cell–cell and cell-basement interactions, acquire mesenchymal characteristics including a fibroblast-like morphology, a stress-fibre cytoarchitecture and increased migratory capacity [1–4]. First described during early embryogenesis [5], it is now widely recognised that EMT, and the reverse process of mesenchymal to epithelial transition (MET), occur widely not only throughout development [6–8] but also in the adult, facilitating key processes such as wound healing [9] and driving pathologies including fibrosis [10–12] and cancer metastasis when inappropriately regulated [3, 13–16].

EMT, or Epithelial-Mesenchymal Plasticity (EMP) as it is also called to reflect its reversible and dynamic nature, is often described as being regulated via a small number of core transcription factors (TF) and by extension, a select group of microRNAs (miRNAs) with which these TFs participate in regulatory feedback interactions [17, 18]. Across the literature, however, hundreds of different TFs and miRNAs have been individually implicated as driving EMT/MET. That is to say, the individual manipulation of hundreds of separate TFs or miRNAs result in a reported EMT or MET phenotypic change as indicated by cell morphology, the altered expression of EMT marker genes (such as the E-cadherin to N-cadherin switch) and changes to the migratory and invasive capacity of cells.

In this review, we seek not to simply catalog these studies (indeed their vast number would make such a task impractical), but to ask a more fundamental question of is it really feasible there exist so many regulators of a biological process such as EMT? If many hundreds of direct regulators seem implausible, why have so many been implicated? Alternately, if hundreds of regulators are a biological reality, why is such complexity required and what challenges might this pose for attempts to manipulate EMT as a therapeutic strategy?

## To EMT or not to EMT: a question of definition?

One of the confounding factors that likely contributes to the vast array of reported EMT regulators lies with the very definition of EMT itself. For example, is it sufficient to ascribe EMT/MET based upon key marker gene expression alone or are phenotypic changes also required? If so, what genes and what effects constitute a minimum threshold? This question is made all the more difficult by the growing realization that EMT is not a separation between two alternate states, but rather a continuum of partial or hybrid EMT states with the intermediate nature of the state itself central to the phenotype [19–24].

A hallmark of EMT are morphological and cytoskeletal changes that alter cell–cell and cell–matrix contacts. These include the downregulation of key genes such as CDH1 (E-cadherin) and CRB3 (Crumbs3), contributing to the loss of adherens and tight junctions, respectively [15]. The loss of E-cadherin is often accompanied by “cadherin-switching” [25], facilitating motility through the upregulation of N-cadherin which mediates more flexible cell–cell contacts. EMT further enhances motility by the cytoskeletal rearrangements that promote focal adhesions (crosslinking actin filaments to integrins) and invadopodia (where matrix metalloproteases degrade an extracellular matrix otherwise unconducive to motility) [15]. These events are thought to underlie metastasis, with which EMT has been extensively linked. Indeed, tumour cells at both the invasive front [26–31] and within the circulation [32–37] often lose epithelial and/or gain mesenchymal markers and the expression of EMT-promoting TFs often correlates with poor clinical outcome [38, 39] and drives metastasis in animal models [40–43]. It may seem reasonable to require such evidence to be presented when claiming an EMT-regulatory role for a new gene or stimulus, however, the causative link between EMT and metastasis remains controversial [16], the morphological and cytoskeletal appearance of cells undergoing EMT varies widely and the inference that changes in the (often two-dimensional) motility of cells in vitro reflects events in vivo is problematic [44]. Further, the different phenotypic outcomes of EMT have been expanded beyond the traditionally reported effects on morphology and motility and into areas including stemness, chemoresistance and immunosuppression [2, 13]. Outcomes, however, are not universal, with specific phenotypes being more or less prominent depending upon the context.

Consistent with the breadth of EMT-associated phenotypes is the magnitude of the underlying transcriptional changes. Typically, the expression of thousands of genes is altered between epithelial and mesenchymal states, and though efforts have been made to deduce a core EMT signature [45–50], even among very well-established drivers of the process there is significant variation between different models. Another inherent difficulty relying upon marker genes is the assumption that changes in the expression of a subset of genes are providing a readout of a wider EMT process. This is especially problematic given the multiple processes that are associated with EMT and the prevalence of hybrid states.

These issues have led to the recent publication of a consensus statement on behalf of the EMT International Association (TEMTIA) [4], which aims to improve guidelines and definitions for EMT researchers in which it was recommended a combination of molecular markers and cellular changes should be required to define EMT. The nature of the markers and cellular changes required to demonstrate EMT/MET, however, are impossible to codify given the broad spectra of phenotypic outcomes and the inherent variation between cells in the genes that drive these changes. As such, defining rigid minimum criteria that one must meet to demonstrate EMT would seem impossible which in turn leaves open the door to claims of genes being “EMT regulators” when they actually regulate narrow aspects of EMT or regulate largely EMT-independent processes that nevertheless overlap or fall within the wider EMT realm. How many aspects of EMT must a gene regulate to be classed as an “EMT regulator” is, therefore, an open question.

Experimental design must also be taken into account when assessing the quality of any given study, especially if that study relies upon single or poorly controlled siRNAs or supraphysiological levels of expression. Even with these caveats, however, it remains true that for hundreds of regulatory TFs, miRNAs and lncRNAs, claims are made of their regulation of EMT, citing as evidence both marker genes and phenotypic changes and employing both exogenous expression and endogenous inhibition to do so. We contend therefore that questions of definition or quality of study are insufficient to dismiss the bulk of EMT/MET regulators that are reported, which in turn posits the question, why are there so many regulators of EMT?

## EMT: interconnected layers of complexity

### EMT inducing stimuli

EMT is induced when epithelial cells encounter specific signals, the best studied of which being the TGFβ proteins (TGFβ1,2,3)—a subset of a wider TGFβ superfamily that also includes bone morphogenic proteins (BMPs), growth differentiation factors (GDFs), activins and inhibins [51]. Many of these promote EMT in various developmental contexts including mesoderm formation [52], heart development [53, 54], neural crest delamination [55] and palate fusion [56]. Both the TGFβs and BMPs also promote fibrosis within the lung [57], liver [58] and kidney [59] and have been widely associated with enhancing plasticity and invasiveness during cancer dissemination [15]. Whilst being the most extensively studied, TGFβ is but one of the dozens of EMT-inducing stimuli, including other growth factors, cytokines and ligands that initiate signaling events through the binding and activation of cell-surface receptors. Prominent examples include the epidermal growth factor (EGF) [60], hepatocyte growth factor (HGF) [61], fibroblast growth factor (FGF) [62], vascular endothelial growth factor (VEGF) [63], insulin-like growth factor (IGF) [64], inflammatory mediators such as IL-8 [65] and ligands activating Notch [8, 66], Hedgehog [67] and Wnt [68, 69] signaling pathways. Additionally, EMT can be stimulated via non-growth-factor stimuli including hypoxia [70, 71], mechanical stress [72] and the metabolite oxalate [73].

### Core EMT-regulating TFs

The large number of EMT-inducing stimuli initiate gene expression programs that involve and are driven by a broad array of TFs. Direct repression of the CDH1 gene (encoding E-cadherin) by SNAI1 was initially identified as a mechanism to drive EMT [74, 75]. Additional TFs have since been identified that also promote EMT, at least in part through direct CDH1 repression. These include the Snail family member SNAI2 [76], the ZEB family TFs ZEB1 and ZEB2 [77, 78] and a host of additional TFs including TBXT [79], E47 [80] and KLF8 [81]. EMT promoting TFs that work via mechanisms independent of direct CDH1 transcriptional repression are also established with better characterized examples including TWIST1 and TWIST2 [82], PRRX1 [83], GSC [84], TCF4 [85], SIX1 [86], FOXC2 [87] and SOX4 [88]. These differing mechanisms result in differing properties. The SNAIL and ZEB TFs for example are potent suppressors of the epithelial phenotype (consistent with direct suppression of CDH1), whilst TWIST and PRRX1 are more potent mesenchymal inducers [2]. Working in opposition are other TFs that enforce an epithelial phenotype including OVOL1 and OVOL2 [89], GRHL2 [90], p53 [91], ELF5 [92], FOXO3 [93] and FOXA1 [94].

The listed TFs, however, only represent a small number of those that have been directly implicated as driving either EMT or MET. Some of these are only mentioned sporadically whilst others are referenced in the majority of EMT studies. Of the best characterized TFs, it is the Snail, Zeb and Twist families that have become recognized as “core” EMT drivers which orchestrate widespread gene expression responses, including supporting the expression of each other. For example, TGFβ promotes the rapid upregulation of SNAI1 and SNAI2 in a manner dependent upon the SMADs and HMGA2 [95, 96] which in turn upregulates ZEB, the expression of which can then be maintained by autocrine TGFβ production which promotes mesenchymal stability [97]. Similarly, both SNAI1 and TWIST1 co-operate in the regulation of ZEB1 to promote EMT [98]. There are, however, many instances of non-redundant functions where the suppression of a single EMT-promoting TF is sufficient to block or severely curtail EMT and metastasis in experimental models without compensation by other core TFs [40, 41, 99–102]. There are also suggestions that specific sub-roles exist between core EMT TFs. For example, SNAI1 and/or SNAI2 are specifically associated with the resistance to chemotherapy [103, 104] whilst ZEB1 prevents apoptotic cells death [105–107]. There are even examples of family members playing opposing roles depending upon context [108]. For example, ZEB1 promotes the initiation and metastatic progression of melanoma which is supported by TWIST1, whilst ZEB2, supported by SNAI2, acts as a melanoma tumour suppressor [109]. The ZEBs also have opposing roles in osteoblast growth and differentiation [110] whilst SNAI1 and SNAI2 differentially regulate stemness and oncogenesis in cells of mammary and thyroid origin [111].

Thus, even at the most basic level of gene regulation in EMT—the actions of a handful of core mesenchymal-promoting TFs, complexities underlie the differential functions of these related family members. The explanations for this are various. For example, the unique targeting of genes resulting from small differences in their “E-box” DNA recognition motifs or the requirement for single or paired E-boxes at varied spacing on account of the positioning of the single (SNAIL) or paired (ZEB, TWIST) DNA-binding zinc fingers. Their regulation of genes is also influenced by their capacity to act as either transcriptional repressors or activators, which can alternate depending upon the cohort of cofactors with which they interact [112]. One such example was recently demonstrated with ZEB1. Although best characterized as a transcriptional repressor via direct binding to DNA at E-box motifs, ZEB1 can be recruited to a co-activator complex through interaction with the AP1 factors FOSL1 and JUN and the Hippo pathway TF, YAP1 [113]. Both actions of ZEB1 functionally synergise as ZEB represses epithelial genes and tumour suppressors and promotes the expression of oncogenes and EMT inducers including TGFβ1 and PTPN14, both genes that encode proteins capable of initiating EMT in their own right [114, 115].

### Co-regulatory relationships between TFs and miRNAs

From the early days of miRNA network biology, it was reported that TFs were enriched among miRNA-predicted targets [116, 117] and TFs frequently form “hub” or key nodes within miRNA regulatory networks [118–120]. Such networks include both feedforward loops, whereby either the TF or miRNA regulate the other whilst both regulate a common downstream target, and feedback inhibition where the TF and miRNA both directly suppress the expression of the other at the transcriptional and post-transcriptional levels, respectively [121].

Gene circuits of this nature are widespread beyond EMT as they may reduce signaling noise [122, 123] and establish mutually exclusive phenotypic states; the most obvious example represented by the phenotypic balance that exists between the epithelially expressed miR-200 family of miRNAs and the mesenchymal promoting ZEB TFs which exist in a direct negative feedback relationship [124, 125]. Similar well-established feedback mechanisms exist between SNAI1 and miR-34 [126], SNAI1 and miR-203 [127] and SNAI2 and miR-200 [128]. Additional, more complex gene regulatory networks (GRNs) also exist. For example, the sequentially expressed EMT-promoting TFs SNAI1 and PRRX1, negatively regulate each other via a feedback loop involving miR-15, where SNAI1 directly represses PRRX1 transcription, whilst PRRX1 transcriptionally activates miR-15, a SNAI1-targeting miRNA [129]. Feedback motifs such as these exist within complex webs of TF:TF and TF:miRNA interactions. One study to illustrate this found that siRNAs targeted against 117 different TFs blocked TGF-β induced EMT in NMuMg cells (as determined by high throughput microscopy assessing cytoskeletal hallmarks of EMT—actin stress fibres, focal adhesions and fibronectin patches) [130]. Coupled with a similar (though more limited) screen for miRNAs that influence EMT in the same model system [131], a connected network of 46 TFs and 13 miRNAs were suggested to regulate EMT, each linked within a web of predicted positive and negative feedback loops that included 4 particularly important TF signaling hubs (ZEB1, TEAD2, FOSL2 and SOX4).

One of the key features of miRNAs is their capacity to simultaneously regulate large cohorts of genes, afforded by the short, and therefore frequently occurring, length of sequence complementarity through which they interact with their targets [132]. For example, miR-200 targets networks of genes associated with the dynamic regulation of the cytoskeleton which is a key component of EMT [133–135] and targets networks of genes downstream of the TGFβ and EGF receptors, perhaps the two best established EMT-inducing stimuli [136]. When coupled with the direct regulation of key transcriptional regulators it is through this “two-punch” mechanism (direct regulation of both the transcriptional regulators and the downstream non-TF network components) that miRNAs exert profound regulatory effects on gene expression. It is worth noting the extent to which the influence of miRNAs is mediated not just directly through their primary target genes, but also indirectly via the targets of the TFs that the miRNAs directly regulate. For example, examining the profile of Dicer-knockout fibroblasts (in which miRNAs are globally depleted), revealed a predominant effect on gene expression at the transcriptional level, both in terms of the number and degree of gene expression changes [137]. This has also been demonstrated specifically with regard to EMT, where both the expression or inhibition of miR-200 resulted in a series of co-ordinated transcriptional responses that were central to MET/EMT and that were likely the result of not only the direct regulation of ZEB but other TFs as well [136].

### Alternative splicing

**Fig. 1:**
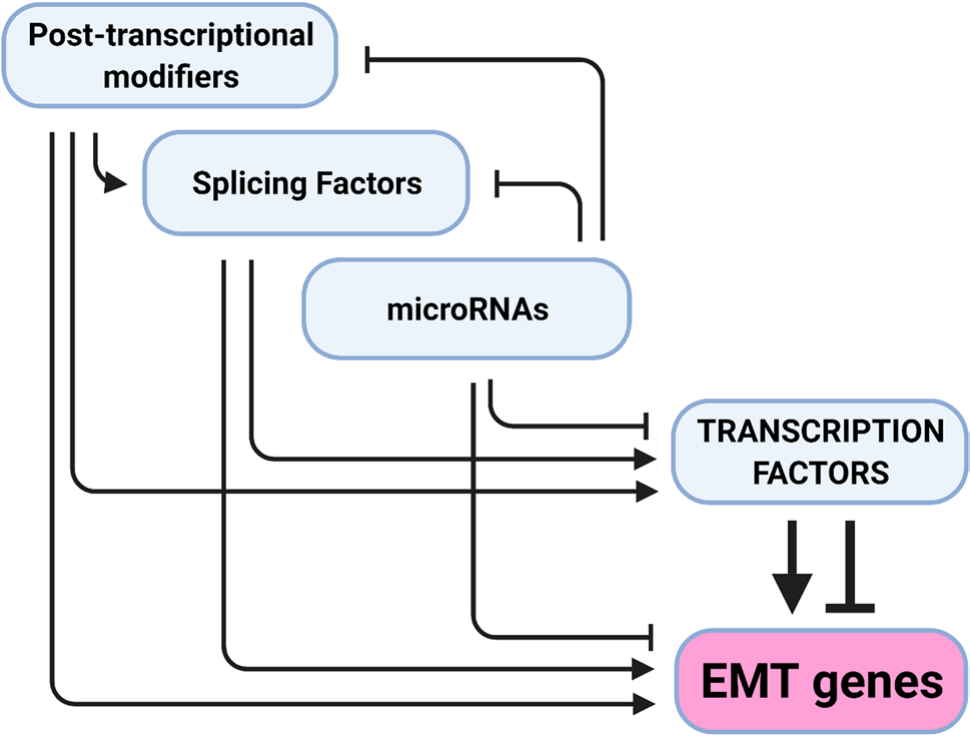
Multiple levels of gene regulation coalesce during EMT

Although not the primary focus of this review, it is worth noting that the complex networks of TFs and miRNAs that regulate both each other and various EMT-associated genes are themselves embedded within additional mechanistic levels of regulation comprising alternative splicing, translational regulation and post-translational protein modification that affects protein stability and subcellular localization (Fig. 1).

Epithelial and mesenchymal cells show distinct alternative splicing patterns [138], regulated by splicing factors whose expression are controlled by EMT-TFs and miRNAs. An epithelial splicing pattern is primarily enforced by two paralogous RNA binding proteins, ESRP1 (epithelial-splicing regulatory protein 1) and ESRP2 which recognize a core UGG motif to guide exon skipping or inclusion depending upon whether the binding site is up- or downstream of the splicing junction [139–141]. In so doing, the ESRPs directly drive several hundred epithelial-specific splicing events, including the production of a shorter isoform of CTNND1 (p120 catenin) that promotes an epithelial phenotype by stabilizing E-cadherin at the cell membrane [142]. During EMT, the ESRP genes are directly downregulated by SNAI1, ZEB1 and ZEB2 [143, 144] whilst other RNA-binding proteins such as QKI (quaking), MBNL1 (muscleblind-like splicing regulator 1) and RBFOX (RNA-binding Fox-1 homolog) promote mesenchymal-specific splicing events [138, 139, 145–147] and guide circular RNA formation [148]. The expression of both QKI and RBFOX1 for example have direct effects on the splicing of genes enriched for EMT-associated processes such as cell motility, the cytoskeleton and stem cell fate determination and guide specific splicing events of consequence to EMT progression [149]. Examples include the production of a shorter isoform of CD44 that is required to activate AKT during EMT [150] and an exon skipping event that results in the redistribution of FLNB (Filamin B) into the cytoplasm and the subsequent release of the EMT-promoting TF FOXC1 [149].

As with other aspects of EMT, complicated interconnected relationships exist between splicing regulators. For example, the epithelial splicing factor RBM47 (RNA binding motif protein 47) both promotes and antagonizes specific alternate splicing events driven by ESRP [146]. On a similar note, SRSF1-regulated mesenchymal splicing of the Ron tyrosine kinase receptor and the Rac1 GTPase is antagonized by opposing events mediated by the SRSF3 and hnRNPA1 splicing factors in epithelial cells [151, 152]. To add even further complexity, gene regulation during EMT can also operate at the RNA level independently of miRNAs or splicing. PTBP3 (polypyriminine tract binding protein-3) for example promotes EMT through the direct binding and stabilization of the ZEB1 mRNA [153].

### Translational regulation

In addition to the regulation of transcription and the influence of microRNAs and alternative splicing on those transcripts, general translational regulatory mechanisms also have a substantial impact upon EMT. YB1 (Y-box binding protein 1) generally suppresses cap-dependent translation on mRNAs but facilitates the cap-independent translation of a subset of mRNAs, including EMT-promoting TFs (SNAI1 ZEB2, TWIST1, LEF1 and TCF4) [154] which are preferentially translated from internal ribosome entry sites (IRES) formed from stem-loop structures within their 5’UTRs [154, 155]. Depletion of the translation initiation factor eIF3e similarly promotes the preferential expression of key EMT TFs [156].

Cap-independent translation can be further aided by N6-methyladeosine (m6A) base modification, with m6A modified mRNAs able to be translated in the absence of eIF4E [157, 158]. m6A modification is the most abundant RNA modification, with functional effects regulated by dynamic interactions among associated methyltransferases (“writers”), demethylases (“erasers”), and binding proteins (“readers”) [159]. TGF-β-induced EMT is inhibited in cells that have reduced expression of the METTL3 “writer”, with lower m6A modification of the TGF-β1 mRNA resulting in lower TGF-β protein production and reduced secretion [160]. Reduced METTL3 expression also downregulated SNAI1 among a group of mRNAs that become m6A-modified during EMT and which is enriched for transcripts encoding proteins related to migration and adherens junctions [161]. In the case of SNAI1, widespread m6A modification throughout the translated region enhanced translational elongation via interaction with YTHDF1, a “reader” that recognizes m6A modified mRNA and recruits the eEF2 translation elongation factor.

### Post-translational modification

EMT TFs are also subject to regulatory mechanisms at the post-translational level of which the best characterized is SNAI1, an unstable protein that is rapidly induced in many EMT systems [162]. SNAI1 phosphorylation by CK1 (casein kinase 1), CK2 or DYRK2 (dual-specificity tyrosine-phosphorylation-regulated kinase) primes phosphorylation by GSK3β (glycogen synthase kinase 3β) to create a recognition site for βTCRP, an E3-ubiquitin ligase that leads to SNAIL degradation [163, 164]. Alternately, the phosphorylation of SNAI1 by PAK1 (p21 activated kinase 1) and ATM (ataxia telangiectasia mutated) kinases increase protein stability [14, 165, 166]. Other post-translationally regulated mechanisms of proteasomal degradation have been reported for SNAIL and other core EMT TFs [167–176]. Phosphorylation can also guide sub-cellular localization. PKD1 (protein kinase D1)-mediated phosphorylation of SNAI1 for example promotes its nuclear export and mutation of the SNAI1 phosphorylation site promotes mesenchymal-like features [168]. In contrast, the nuclear phosphorylation of a different site in SNAI1 by LATS2 (large tumour suppressor kinase-2) promotes EMT by increasing its nuclear retention and stabilization [177]. PC2 (Polycomb protein 2)-mediated sumoylation of ZEB2 on the other hand does not affect protein localization, but instead affects transcriptional activity with sumoylation disrupting the interaction of ZEB2 with the CtBP co-repressor, thus relieving repression of the CDH1 promoter [178].

### A case study in complexity: ZEB1

There are two aspects to the notion of extensive, interconnected gene regulatory networks. One is illustrated by the discussion above and the sheer volume of different regulators that have been implicated in the process. Indeed, if the criteria for EMT/MET are taken as a change in marker gene expression along with a phenotypic change to cell morphology or motility after perturbation of the levels of an EMT regulator, at least 300 different TFs and miRNAs have been reported to regulate this process. The other aspect of complex regulation is the multiple levels at which any one of these regulators are connected to others within the network. Thus, examination of the true number, roles and significance of EMT regulators is informed by both the number of participant genes and by their interconnectedness (which operates across different levels of gene expression). As a means of illustrating the complexity of regulation and feedback mechanisms that operate within EMT, we will focus specifically on ZEB1 and examine its regulation by other TFs and non-coding RNAs. In so doing, however, we stress that the situation with this gene is not necessarily more complicated than is the regulation of many other genes within an EMT system, thus highlighting the complex web of control that has evolved.

### Regulation of ZEB1

The ZEB1 and ZEB2 proteins, along with members of the SNAIL and TWIST families, constitute core components of the EMT-regulatory network, directly regulating a transcriptional response through interacting with paired E-box motifs within the regulatory regions of genes encoding components of adherens and tight junctions, desomosomes and intermediate filaments [179, 180]. Via the recruitment of histone deacetylases, DNA methyltransferases and components of the SWI/SNF and CtBP co-repressor complexes [181, 182], the ZEBs typically mediate gene repression. However, and in contrast to other core EMT TFs, the ZEBs can also mediate transcriptional activation, recruiting P/CAF and p300 co-activator complexes to promote the expression of mesenchymal genes including as N-cadherin, vimentin, fibronectin and matrix metalloproteases. Conversion from acting as repressors to activators of transcription is brought about via interaction with other proteins including β-catenin and YAP1, effectors of the Wnt and Hippo signaling pathways, respectively [183, 184].

Over the past decade or so, the number of known EMT-regulatory TFs has grown dramatically, though a central pro-mesenchymal function for the ZEBs continues to be reported, often within the context of the miR-200:ZEB co-regulatory loop. Here, members of the miR-200 family (epithelial enforcers) directly bind and downregulate the stability and translation of the ZEB mRNA, whilst the ZEB proteins directly bind and repress the promoters of both miR-200-encoding genomic loci [114, 124, 125, 185]. Illustrating the complexity of the regulation of EMT, even in the context of a single—albeit very important—gene, is that to date, no fewer than 62 different miRNA families have been reported to directly target ZEB1 as indicated by the miRNA-responsive expression of a reporter gene (typically luciferase) fused to the ZEB1-3ʹUTR (Table 1). Such an experiment, considered a gold-standard in the field of miRNA research, is often accompanied by additional measurements of ZEB1 mRNA and protein expression after miRNA perturbation. Even accounting for the reliance on miRNA over-expression in a number of these studies, it is still clear that a gene such as ZEB1 (as well as ZEB2 and members of the SNAIL and TWIST families) are subject to extensive regulatory control.

**Table 1:** List of miRNAs that have been experimentally demonstrated to target ZEB1 (as demonstrated by ZEB1-3ʹUTR reporter assay)

microRNA	miR modulation	MRE mutation	PMID
miR-10	Overexpression	Y	25896413
miR-101	Overexpression	Y	24677166
miR-101	Overexpression	Y	25808945, 27429852
miR-1199	Overexpression	Y	29079737
miR-1236	Overexpression	Y	24573236, 31799668
miR-124	Overexpression	Y	31793989
miR-126	Overexpression	Y	28379605, 31007650
miR-127	Overexpression	Y	28636101
miR-1271	Overexpression	Y	26940738, 31695412
miR-128	Overexpression	Y	25921099, 29329360, 31352238
miR-129	Overexpression	Y	32210737
miR-130	Both	N	22847613
miR-130	Overexpression	Y	28754469, 31207321
miR-136	Overexpression	Y	30203524
miR-139	Overexpression	Y	25833697, 26022123, 32641995
miR-140	Overexpression	Y	29416674
miR-142	Overexpression	Y	23342264, 30092578
miR-143	Overexpression	Y	28543721
miR-144	Overexpression	Y	26191328
miR-150	Overexpression	Y	25090005, 28781686
miR-150	Overexpression	N	32013135
miR-1786	Overexpression	N	24763497
miR-183/ ~ 96	Overexpression	Y	24277930
miR-186	Overexpression	Y	29325758, 29475118, 32388910
miR-194	Overexpression	Y	32042767, 32862492
miR-199	Overexpression	Y	31705138
miR-199	Both	Y	32046378
miR-199	Inhibition	N	29959879
miR-200	Both	Y	18376396, 18381893
miR-200	Overexpression	N	18411277
miR-204	Overexpression	Y	27020592, 28861151, 30107990
miR-216	Overexpression	Y	24958806
miR-217	Both	Y	30212709
miR-217	Overexpression	Y	30794031
miR-223	Overexpression	Y	28981085, 31760895
miR-223	Both	N	27744452
miR-23	Overexpression	Y	29778425
miR-23b	Overexpression	Y	23844063
miR-27	Overexpression	Y	29102917
miR-27	Overexpression	Y	30549040
miR-28	Overexpression	Y	30058089
miR-3129	Overexpression	Y	30615851
miR-33	Overexpression	Y	25868853, 26459797, 31401160
miR-34	Overexpression	Y	29102917
miR-340	Overexpression	Y	27036021
miR-342	Overexpression	Y	29495972, 30061949
miR-3622	Overexpression	Y	28498363
miR-3666	Overexpression	N	26383522
miR-381	Overexpression	Y	29295724, 29523223
miR-409	Overexpression	Y	27079864, 30448056, 30846940
miR-431	Overexpression	Y	26697292
miR-432	Overexpression	Y	33178684
miR-448	Overexpression	Y	29323713, 29368542
miR-451	Overexpression	Y	32335297
miR-455	Overexpression	Y	26801503, 29216394
miR-455	Both	Y	31492753
miR-4652	Overexpression	Y	30849635
miR-4677	Overexpression	Y	31173403
miR-484	Overexpression	Y	28286418
miR-508	Overexpression	Y	29374066, 30338806, 30988768
miR-5590	Overexpression	Y	31570691
miR-574	Overexpression	Y	29755127, 30917930
miR-590	Overexpression	Y	26556542
miR-591	Overexpression	Y	23807165
miR-601	Overexpression	Y	32694942
miR-641	Overexpression	Y	30588009
miR-644	Overexpression	Y	30808676
miR-652	Overexpression	Y	26498682
miR-655	Overexpression	Y	23765923
miR-665	Overexpression	Y	31573758
miR-708	Overexpression	Y	29575368
miR-708	Overexpression	N	31632515, 31962101
miR-873	Overexpression	Y	30455125, 31579087
miR-873	Overexpression	N	33133224

As previously highlighted, complexity within a regulatory network such as that controlling EMT has multiple levels, first and most obviously the simple number of regulators that have been ascribed this role (such as the > 60 miRNA families reported to directly regulate ZEB1). Complexity is further demonstrated by the frequent participation of ZEB1 in mutual co-regulatory loops with these miRNAs, and by the close association of ZEB1 and these miRNAs with other TFs and miRNAs that themselves also play central roles, building networks motifs of increasing complexity. The reporting of such higher network architecture may be contained within a single study, though the practical limitations of experimental methodologies limit the scope to which this is possible. The heavily studied nature of the field, however, enables a picture of the complex relationships within the network to emerge when considering multiple reports examining smaller subnetworks, motifs and the individual relationships between genes of interest.

**Fig. 2:**
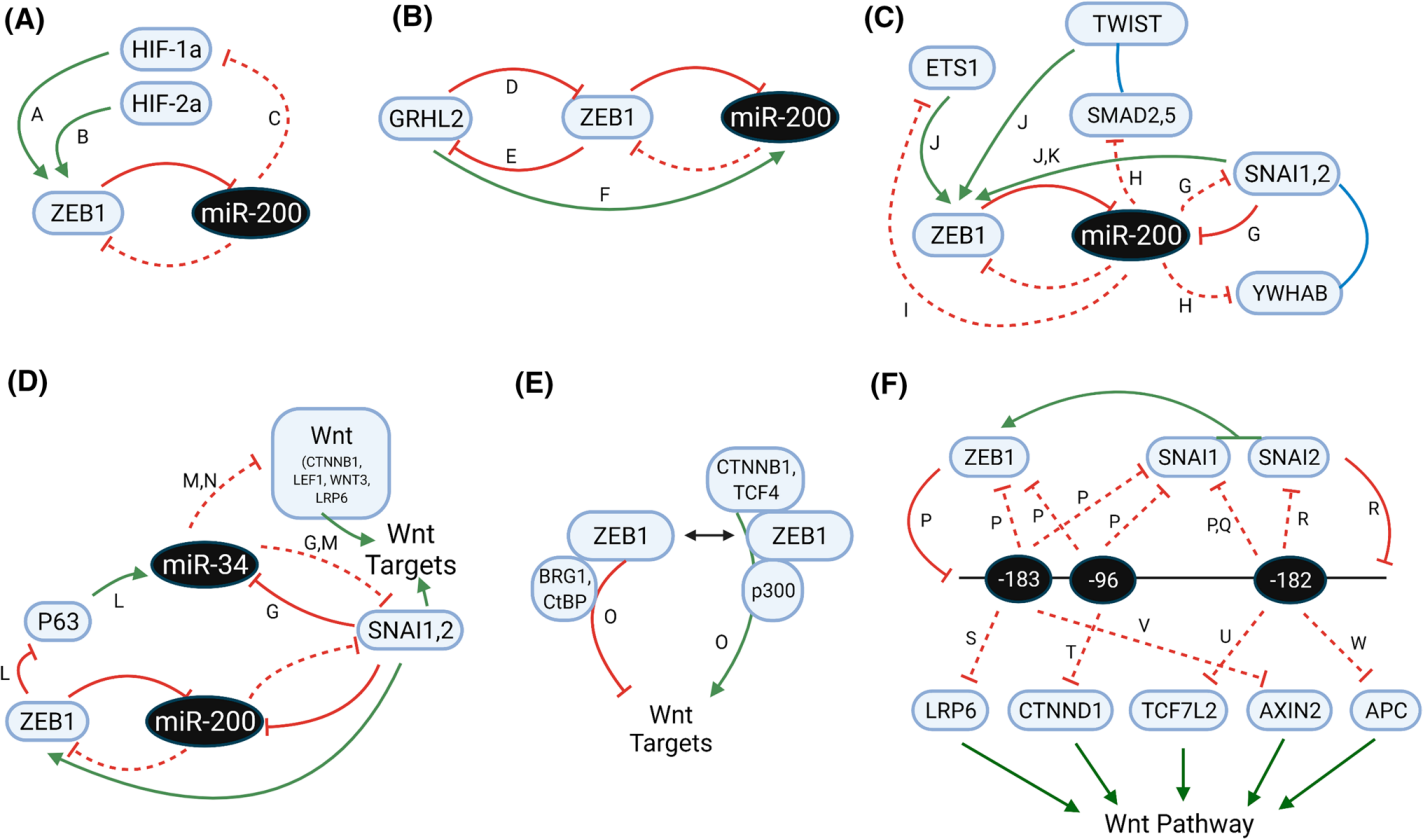
Regulatory loops incorporating ZEB1 into larger networks involving other TFs and miRNAs. Letter annotations denote supporting PMIDs (A = 26057751; B = 19662677; C = 28899657; D = 22379025; E = 23943797; F = 26887971, 26933170; G = 22370643; H = 25798844; I = 21081489; J = 21317430; K = 21593765, 29259250; L = 22850877; M = 22024162; N = 22421157, 22045851; O = 22080605, 26387539; P = 24277930, 25394902; Q = 23354685; R = 27894095; S = 24289859; T = 31913290; U = 29733821; V = 30070321; W = 31938296)

In Fig. 2, such examples are highlighted. In some such examples, the operation of the miR-200:ZEB loop itself can be modulated by other TFs to either bias ZEB (mesenchymal) or miR-200 (epithelial) expression. For example, by directly binding and transactivating the ZEB1 promoter, the Hypoxia Inducible Factors (HIF1a and HIF2a) potentiate EMT (Fig. 2a), which is counteracted by the direct suppression of the HIFs by miR-200 [186, 187]. Alternately, the epithelial enforcer Grainyhead-Like 2 (GRHL2) potentiates MET, promoting the miR-200 arm of the co-regulatory loop by directly promoting transcription of the miR-200 genes, whilst also participating in a direct negative transcriptional loop with ZEB1 (Fig. 2b) [90, 188–191]. ZEB1 is further modulated by the additional co-regulatory loops in which miR-200 participates. This includes direct reciprocal negative feedback between miR-200 and the SNAIL TF family, whilst the SNAILs, TWIST1 and ETS1 all directly bind and transactivate the ZEB1 promoter. MiR-200 further modulates this regulation, directly targeting ETS1 and both SMAD2/SMAD5 and YWHAB/YWHAG, co-factors for both TWIST and SNAIL, respectively (Fig. 2c) [192, 193]. SNAI1 also participates in a co-regulatory negative feedback loop with miR-34, itself indirectly controlled by ZEB1 via ZEB-mediated transcriptional repression of the miR-34 transactivator, p63 [194]. miR-34 directly targets multiple components of the Wnt signaling pathway, with which both SNAI1 and ZEB1 have direct linkages via the interaction with the Wnt pathway co-activator, β-catenin (CTNNB1) and by direct transcriptional activation by the β-catenin/TCF4 complex (Fig. 2d) [126, 184, 195–198]. Indeed, this interaction converts ZEB1 from a direct repressor of Wnt pathway targets to an activator as ZEB1 binds TCF4 and swaps co-repressor (Brg1, CtBP) for co-activator (p300) proteins (Fig. 2e) [183]. Perhaps an even more complicated subnetwork applies to the miR-183 ~ 96 ~ 182 cluster, with both ZEB1 and SNAI1/SNAI2 directly repressing transcription of the miRNA host gene whilst multiple miRNAs within the cluster target these same TFs. Interestingly, the miR-183 ~ 96 ~ 182 cluster is also directly transcriptionally activated by B-catenin/Wnt signaling, resulting in multiple complex outcomes downstream as the miRNAs variously target both Wnt pathway activators (LRP6, CTNND1, TCF7L2) and repressors (AXIN2, APC) (Fig. 2f) [199–206].

Examples such as those described above are not intended as an exhaustive catalog of ZEB-associated EMT pathways. Indeed, the number of these interactions would make such a task almost impossible. What it does showcase, however, is that even if one discounts a large volume of studies that are reliant upon over-expression or those in which EMT-associated outcomes are demonstrated by limited means, tremendous complexity is still apparent—even if one just considers a single EMT regulator like ZEB1. This same conclusion would also be drawn from an in-depth focus into the regulation of SNAI1, SNAI2, TWIST1 and any number of dozens of other key TFs for which the sum of evidence supporting their EMT-regulatory capacity is overwhelming.

## EMT: why complexity is a necessity

What is known about any system is proportional to the time devoted to its study. Thus, given the importance of EMT to both development and pathology, enormous effort has been dedicated to understanding EMT regulation and function. Even with this in mind, however, it is clear that tremendous complexity has evolved around EMT, illustrated not just by a large number of regulators, but also via the multiple levels of gene regulation at which they operate. By necessity, such complexity is largely ignored in individual studies that seek to uncover roles for specific genes in specific contexts. Here, we consider why such complexity is necessary.

### The ubiquity of EMT/MET: multiple contexts

During development, a broad range of stimuli are deployed at different times and in different sites to guide EMT in a diverse range of cells. Therefore, it is perhaps not surprising that a large number of sensors, effectors and modulators have evolved to facilitate EMT across the multiple contexts in which it is activated. The diverse nature of responsive cells is seen by the fact that EMT/MET-like processes, often driven by the same core EMT TFs, still promote the aggressiveness of cancers in tissues of non-directly epithelial or mesenchymal origin such as gliomas (originally derived from primitive neuroectoderm), sarcomas and haematological malignancies (derived from muscle and bone, originally of the embryonic mesoderm) [207].

Another complexity is that EMT itself varies widely across different contexts, both in terms of the range of possible phenotypic outcomes and in the nature of the underlying transcriptomic profiles. For example, the majority of genes that were differentially expressed between cell lines derived from the lung, kidney and breast were unique in response to the same EMT stimulus (TGFβ + TNFα) [208]. Transcriptomic profiling of single-cell lines of ovarian, prostate, breast and lung origin in response to 3 different EMT inducers (TGFβ, EGF, TNFα) also showed little overlap of responsive genes, both between the same stimulus across different cells and between different stimuli in the same cell [209]. It is therefore clear that not only are there divergent EMT pathways between cells, but that multiple EMT pathways are operable in the same cell, triggered by ligands binding different cell surface receptor kinases. Additionally, the promoters of key genes such as CDH1 [210] and ZEB1 [211] can simultaneously display both repressive and active marks, creating a poised bi-valent state which allows rapid on–off cycling and likely contributes to EMT reversibility. Complexity therefore broadly arises from several sources: the requirement for cells with a differing gene expression landscape to still be responsive to EMT, and to facilitate different phenotypic outcomes tailored to the specific context and nature of the stimulus.

### Flexibility–reversibility and partial EMT phenotypes

Historically, EMT has been viewed as a binary process whereby cells undergo transformations between epithelial and mesenchymal states, as often defined by the gain and loss of select epithelial (E-cadherin) and mesenchymal (N-cadherin, vimentin) markers. Subsequent mathematical models and biological observations, however, now supported by single-cell RNA sequencing (scRNA-Seq), clearly demonstrate there exists a spectrum of hybrid phenotypes (also referred to as “incomplete”, “intermediate” or “partial” EMT), whereby individual cells co-express both epithelial and mesenchymal markers [212].

Not only have hybrid states been noted across a diverse range of cells, both in cell culture and in vivo (reviewed in [212]), but the existence of a hybrid state itself is of tremendous functional significance as it is tied to the capacity of cells to migrate during both development and cancer and in the promotion of stemness properties. Hybrid E/M phenotypes allow collective cell migration by maintaining adhesion between neighbouring cells whilst decreasing apico-basal polarity, thus increasing the motility of the leading cells. Collective migration is used during embryonic development such as in the branching morphogenesis of the mammary gland or the sprouting angiogenesis of endothelial cells [213, 214]. It is also employed in the adult, both in essential processes such as wound healing [215] and in pathologies for which it has gained particular attention. Fibrotic renal tubular epithelial cells for example display a hybrid EMT phenotype [216–218], as do cells at the invasive front of tumours which corresponds to poor survival across many tumour types [24, 219]. Circulating-tumour cells (CTCs) that are associated with a diverse array of cancers also display a hybrid phenotype [35, 220–223] and the presence of a hybrid state is more closely associated with a poor clinical outcome than are fully epithelial or mesenchymal features [220, 224–227].

Initially, EMT was proposed to increase stemness [228, 229], however, it was subsequently found that cells that become locked in an exclusively mesenchymal state actually lose their stem-like properties [101, 230] and it is in fact the hybrid state that creates a “stemness window” [231, 232]. Cells displaying such hybrid features exhibit increased tumourigenic capacity [233] and the ectopic expression of EMT TFs enhances the formation of secondary tumours upon transplantation [228, 229]. Growing evidence also links the hybrid E/M phenotype with the resistance to therapy [43, 234–237], further suggesting that targeting hybrid E/M cells may be a productive focus for therapeutic strategies.

The mathematical modelling that was initially employed to predict stable intermediate states (reviewed in [238, 239]) has been superseded by single-cell sequencing which suggests that waves of continuous gene expression give rise to a myriad of intermediate phenotypes [219, 240–242]. The capacity of cells to reside within such hybrid states is supported by phenotypic stability factors (PSFs); genes expressed in hybrid E/M phenotypes that counteract the full transition by regulating core EMT regulators [232, 238, 239, 243]. The first such PSFs to be predicted and experimentally validated are OVOL2 and GRHL2, TFs known to induce MET or halt EMT in a context-dependent manner by suppressing several EMT promoting TFs such as ZEB1 with which they form mutually inhibitory loops of regulation [89, 90, 190, 244–247]. Subsequently, the list of factors to be given the PSF designation has grown. For example, miRNAs such as miR-145 and miR-129 have been described as PSFs through the opposing roles they play against ZEB2 [248] and TWIST1 [249] respectively, whilst NRF2 contributes to a hybrid E/M state by suppressing SNAIL [250].

**Fig. 3:**
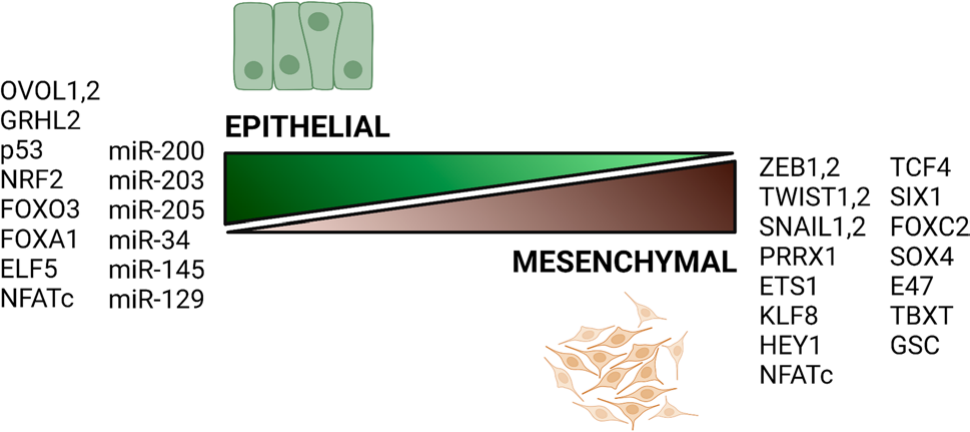
Opposing roles of major EMT-regulatory transcription factors and microRNAs

It may be that certain factors specifically function as PSFs. NFATc for example induces both epithelial (E-cadherin, miR-200) and mesenchymal (ZEB1) genes and thereby stabilises a hybrid E/M phenotype [251]. Given the interdependent nature of gene regulation, however, it could be argued essentially all system components contribute a PSF role. Even E-cadherin (CDH1) for example could be regarded in this manner, sequestering B-catenin which in turn prevents the transcriptional activation of ZEB and thereby, prevents ZEB’s inhibitory role on the CDH1 promoter [252]. We would argue therefore that for the most part, PSFs should be thought of less as specific hybrid maintenance factors, and more simply as components of the large and overarching genetic networks through which the E/M status of a cell is derived as a result of the multitude of opposing signaling outcomes (Fig. 3). This notion of multiple PSFs functioning as small parts within wider networks may also be a more helpful way to approach complex networks, given the tendency in papers to ascribe major consequences to single effectors which both comes from the publication-incentivised over-interpretation of results and the over-simplification of gene regulation that necessarily follows.

Along with the growing number of studies that demonstrate “non-canonical” TFs (such as HEY1, FOXO3 and FOXA1) can also induce EMT independently of the canonical EMT TF core, the number of potential PSFs that interact with them will also grow, further enlarging the complexity of EMT regulation [239]. It is yet unknown to what extent non-core TFs can drive EMT and it may be that their contribution is larger than generally recognised. ScRNA-Seq for example identified the widespread hybrid characteristics of developing intestinal, lung and liver cells despite their very low expression of SNAIL, ZEB and TWIST family genes [240]. By employing additional PSFs to also regulate these regulators, the EMT system further increases its information capacity, meaning the system can reside in multiple distinct states for specific purposes, further fine-tuning traits associated with motility and stemness.

### Noise reduction, buffering and inbuilt safety

The requirement that many types of cells be responsive to EMT is a likely driver of the large number of stimuli that are capable of promoting EMT. Cells, however, are required to balance responsiveness with blocking inadvertent activation, as this could have serious consequences, including promoting the metastasis of cancer. The potential for spurious induction is heightened by the observation that even a common metabolite such as oxalate can promote EMT [73], raising the question of how many other metabolic by-products to which cells are frequently exposed could also act as triggers? In addition to exposure to a broad range of external stimuli, random fluctuations in the expression level of individual genes within the EMT network could also propagate through the system if such gene expression noise impacts upon key targets such as regulatory TFs. If these fluctuations occur around a critical threshold of TF abundance, for example, the noise from initially minor variations could be propagated to result in radical transcriptomic (and phenotypic) changes. The need to buffer such transcriptomic noise necessitates the evolution of more complex regulatory programs and higher scales of network architecture. The size and complexity of the gene regulatory networks (GRNs) associated with EMT reflect the importance of correct regulation of the process, as well as those additional features such as the diversity of contexts in which EMT must operate, the need for reversibility and the specific importance of hybrid states.

The architectures of GRNs are built from circuits and sub-circuits of interacting genes that mediate specific responses [253]. One such example of relevance is the distinction of 5 EMT sub-circuits (controlling basement remodeling, motility, apical constriction, apical-basal polarity and de-adhesion) operational during sea urchin development [254]. Even in this relatively simple system (primary mesenchyme cells; the first cells in sea urchin development to undergo EMT) complex networks exist in which there is the absence of any single master regulatory TF. This is because at least 13 different TFs are required for the completion of EMT, though no single TF is required for each of the 5 sub-circuits [254]. Single-cell sequencing that follows the progression of EMT has also noted waves of gene expression and series of discrete transcriptional events, suggesting EMT is a multistep process even though it presents as a continuous gradient of gene expression without discernable boundaries between hybrid states [209].

As briefly discussed earlier, complex networks include smaller recurring circuits called “network motifs” which can be broadly divided into two categories: feedback and feedforward [255]. Positive feedback loops often underlie developmental switches; for example, a TF promoting its own expression to facilitate an “all or none” outcome. If that same TF, however, were to induce a repressor of its own expression, a negative feedback loop such as this would limit strong changes. Other types of negative feedback, however, are conducive to molecular switches. The reciprocal feedback loop between ZEB and miR-200 is one such example, where a TF directly represses a miRNA that itself targets the TF. Feedforward motifs on the other hand are based on regulators that act both directly and indirectly on their downstream targets. Multiple outcomes are therefore possible depending upon the nature of the motif. Irrespective of specific network motifs, one overarching principal is that GRNs that incorporate positive and negative feedback increase their potential to control the effects of noise, buffering its impact on gene expression [255–258].

The buffering of biological noise, and the capacity to directly modulate the activity of TFs, make miRNAs ideally suited to the regulation of complex processes. This would explain the association of many miRNAs with EMT, well beyond the best-established examples such as miR-200, miR-203 and miR-34. MiRNA-TF motifs are represented in biological networks at a much higher rate than would be expected by chance; both in a directly reciprocal manner and where a TF positively regulates both a miRNA and a target gene that the same miRNA also negatively regulates [121]. This would explain the seemingly contradictory observation that the expression of miRNAs and their targets are often positively correlated [259, 260]. Experimentally, the capacity of a miRNA to buffer noise was demonstrated using an artificial reporter system consisting of an inducible, self-regulatory TF whose expression level controls an on/off “toggle-switch” phenotype, coupled with a miRNA that targets the TF. When present, the miRNA conferred robustness and enabled the cell to maintain its state though when absent, a dramatic increase in protein noise level caused the cell to randomly switch between states [261]. On a transcriptome-level scale, noise from lowly expressed genes is buffered by miRNAs and genes regulated by multiple miRNAs show greater noise reduction [262]. MicroRNAs also participate in the widespread buffering of transcriptomic noise in EMT systems [263] and the 3ʹUTRs from genes with variably active promoters are more frequently targeted by miRNAs than are the 3ʹUTRs of genes of low transcriptional noise [264].

The requirement for multiple direct regulators may also be a function of the contribution of any one regulator, even a core EMT TF, being insufficient in itself to sway the phenotype in isolation. Thus, a comparatively “weak” effect that is mediated by any individual regulator provides an inbuilt safety mechanism, minimizing the genetic noise that could result from dysregulation of any single factor in isolation. Such a model is consistent with the successive waves of TF expression that is reported after exposure to EMT-inducing stimuli, with the expression of later regulators being dependent upon the upregulation of more rapid responders and their co-operative actions.

## Concluding remarks

EMT encompasses a broad range of processes that are measurable by a number of genetic markers and phenotypic outcomes. It is therefore inherently difficult to establish a minimal evidential threshold to define an EMT regulator (despite recent attempts [4]) which allows one to question the importance of many proposed regulators that are yet to be substantiated by multiple laboratories, or for which the evidence is dependent upon exogenous expression or the measurement of limited markers or phenotypes. Even with this in mind, however, it is clear that EMT/MET is subject to regulation by many dozens (and likely hundreds) of different TFs and miRNAs, themselves subject to additional levels of control via splicing and post-translational modification.

Complexity is apparent with the regulation of any genetic process, though may take on special importance with EMT due to several factors. These include the requirement that multiple types of cells during development and in the adult be capable of E/M plasticity, and the hybrid nature of the process itself as cells balance and combine various features of epithelial and mesenchymal states to facilitate specific outcomes. For the researcher, multiple factors must therefore be considered if one is to take a global view of a complex regulatory process such as EMT. For example, how many phenotypes are associated with EMT? What are the tipping points that restrict reversibility and if a reversal of phenotype occurs, are the molecular pathways involved simply reversed? Further, how is the buffering of gene expression balanced with the capacity for phenotypic change when both of these processes are facilitated by a common pool of potential regulators? Mathematical tools to assist the modelling of such complexity are available [265].

By bolting on feedback and feedforward loops (within which TFs drive genetic programs with miRNAs providing a critical regulatory layer), the information capacity of the system (how many states a system can exist in) is increased, facilitating hybrid states whilst providing additional noise reduction and buffering capacity. In this light, many (if not all) regulators can be viewed as phenotypic stability factors (PSFs)—important contributors that at their endogenous levels do not necessarily lock a phenotype at either end of the E/M spectrum, but rather, that balance opposing effects of other regulators as phenotypes are stabilised along an E/M continuum.

## Data Availability

Not applicable.

## Abbreviations

EMT: Epithelial-mesenchymal transition
MET: Mesenchymal-epithelial transition
EMP: Epithelial-mesenchymal plasticity
miRNA: MicroRNA
TF: Transcription factor
GRN: Gene regulatory network
PSF: Phenotypic stability factor
scRNA-Seq: Single-cell RNA sequencing
CTC: Circulating tumour cell
